# Effect of Acid Mixtures on Surface Properties and Biaxial Flexural Strength of As-Sintered and Air-Abraded Zirconia

**DOI:** 10.3390/ma14092359

**Published:** 2021-05-01

**Authors:** Jong-Eun Kim, Yong-Chan Kwon, Sunjai Kim, Young-Bum Park, June-Sung Shim, Hong-Seok Moon

**Affiliations:** 1 Department of Prosthodontics, Yonsei University College of Dentistry, 50-1 Yonsei-Ro, SeodaemunGu, Seoul 03722, Korea; gomyou@yuhs.ac (J.-E.K.); sunjai@yuhs.ac (S.K.); drybpark@yuhs.ac (Y.-B.P.); jfshim@yuhs.ac (J.-S.S.); 2 CLF Dental Laboratory, 227 Moraenae-ro, Seodaemun Gu, Seoul 03695, Korea; ybg5139@hanmail.net

**Keywords:** acid mixture, zirconia, air abrasion, biaxial flexural strength, 3Y-TZP, as-sintered, sulfuric acid

## Abstract

The aim of this work was to evaluate the effects of application time of an acid mixture solution on the surface roughness, phase transformation, and biaxial flexural strength of 3Y-TZP after sintering or air abrasion. For the biaxial flexural strength measurement, 220 3Y-TZP disk-shaped specimens were prepared after as-sintering or air abrasion. The etching solution comprised a mixture of hydrofluoric acid, sulfuric acid, hydrogen peroxide, methyl alcohol, and purified water. The samples were divided into 11 subgroups according to the etching times (Control, 1, 2, 3, 5, 8, 10, 12, 15, 20, and 30 min). The results showed that acid treatment on both as-sintered and air-abraded 3Y-TZP surfaces increased the surface roughness. However, it had no significant effects on the monoclinic phase or flexural strength of as-sintered zirconia. The monoclinic phase and flexural strength of air-abraded zirconia increased sharply after air abrasion; however, they gradually decreased after acid treatment, to a similar level to the case of the untreated surface. Surface treatment with acid mixture increased the roughness, but the lack of increase of monoclinic phase is thought to be because the loose monoclinic particles remaining on the surface were removed through the etching process.

## 1. Introduction

For many years, the metal ceramic crown has been the most commonly used dental crown in aesthetic prosthetic restoration. However, its use has recently declined owing to problems such as its metal framework color, having an opaque shade compared to natural teeth, and patient metal allergies [1,2]. Recently, various high-strength all-ceramic restorative materials with natural tooth shades have been introduced in the market. They are widely used for tooth loss areas of various spans, including multi-unit bridges [2,3,4]. Among these materials, zirconia, especially yttria-stabilized tetragonal zirconia polycrystals (Y-TZP), is known to offer natural tooth shades, wear resistance, and biocompatibility [5,6]. Because of its high strength, it can be manufactured for monolithic restoration [7,8]. Zirconia materials are increasingly being employed on account of advances in computer-aided design/computer-aided manufacturing (CAD/CAM) technology. In addition, because the zirconia prosthesis can be manufactured at a lower cost than conventional crowns, it is used for various applications, such as implant crowns, implant abutments, inlays, and onlays, as well as crowns and fixed partial dentures on natural teeth [9,10].

Although zirconia is chemically and biologically inert, predictive bonding with zirconia is difficult to achieve in clinical applications [11,12]. Surface treatments have been carried out primarily to achieve this bonding, with the aim of cementing zirconia restorations on teeth or implant abutments. In addition, to produce more aesthetically pleasing prostheses, such treatments have been performed to create rough surfaces for building up porcelain on zirconia copings [13]. The air-abrasion method using aluminum oxide or silica-containing particles has traditionally been employed for the surface treatment required in zirconia clinical applications [12,14]. Some studies reported that air abrasion with aluminum oxide particles can increase the shear bond strength with resin cement or the bond with veneering ceramics by roughening the zirconia surface [15,16]. It was additionally reported that air abrasion can remove foreign substances from the surface and improve surface energy and wettability [17,18].

Other studies reported that creating surface roughness using air-abrasion methods on zirconia surfaces is useful and yields good results in bonding; nevertheless, this technique has several limitations. Firstly, air abrasion can cause stress-induced transformation of zirconia ceramics [4,19]. Stabilizers, such as yttrium and ceria, are used to maintain the zirconia in a tetragonal phase, even at room temperature [20,21,22]. However, when tetragonal-to-monoclinic (t-m) phase transformation occurs on account of aging or mechanical stress, such as grinding, milling, or air abrasion, the monoclinic phase increases. Consequently, the surface structure changes or the surface damage causes negative effects on long-term oral use [19,23]. Moreover, the air-abrasion technique is difficult to standardize because it is challenging to achieve consistent results over the entire application surface when applying particles from the nozzle to the zirconia surface [24]. In the case of zirconia surface air abrasion, various conditions are required, such as optimal pressure, particle size, and particle type. Several studies reported inconsistent results on surface roughness, monoclinic phase contents, and flexural strength owing to the t-m phase transformation [25,26,27]. Specifically, the change in mechanical strength when air abrasion was applied to zirconia differed in each experiment, and the results may have affected long-term performance on account of surface damage [28,29,30].

Acid treatments have also been attempted to roughen the material surface and improve the bond to resin cement or porcelain. Surface etching using hydrofluoric acid has mainly been performed on glass ceramics. This approach removes the silica-containing glassy matrix and exposes the crystalline phase [31,32]. However, zirconia is resistant to conventional etching techniques because it is a silica-free ceramic and consists of a high-temperature crystal phase [11,33,34,35]. Nevertheless, some studies reported that zirconia can also be etched. This is possible when increasing the hydrofluoric acid temperature, or when applying hydrofluoric acid to zirconia for a longer time than when applied to glass ceramics [24,36,37,38,39]. The acid treatment can be simultaneously applied to the entire area; thus, the surface treatment is very uniform compared to that under air abrasion [24]. However, many studies on the effect of air abrasion and the etching process on zirconia strength have not yet reported consistent findings. Some reports contend that these surface treatments increase the strength of zirconia [40,41,42]; others suggest that they decrease its strength [41]. In particular, studies on the effects of etching processes on zirconia materials, such as strength and monoclinic-phase contents, lack adequate evidence [43,44,45]. Among the types of acids used for surface treatment, hydrofluoric acid is used to increase the surface roughness of zirconia, whereas sulfuric acid is known to play a role in stabilizing the tetragonal phase when used in zirconia. It is also necessary to verify the effect that can be achieved [46,47].

In order to increase the mechanical interlocking between luting agents or veneered porcelain and zirconia surfaces and to achieve an efficient performance of predictive dental restoration, it is essential to minimize the damage of zirconia surfaces and to increase their roughness, while maintaining their strength and tetragonal phase. The purpose of this study was therefore to evaluate the effects of acid mixtures containing hydrofluoric acid, sulfuric acid, hydrogen peroxide, and other compounds on the surface roughness, phase transformation, and biaxial flexural strength of 3Y-TZP after as-sintering or air abrasion. The null hypothesis of this study is that neither etching using acid mixture nor the combination pretreatment with air abrasion and etching significantly influences the biaxial flexural strength of zirconia surfaces.

## 2. Materials and Methods

### 2.1. Specimen Preparation

**Figure 1:**
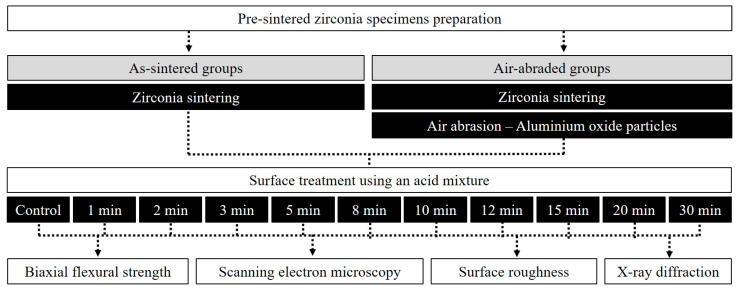
The workflow of surface treatment and analysis for specimens used in this study was conducted as follows.

For the biaxial flexural strength measurement, 220 3Y-TZP with a density of 6.08 g/cm^3^ and a purity of >99% (CAMeleon zirconia block; Neobiotech, Seoul, Korea) specimens were milled (DWX-51D; Roland DGA Corp., Irvine, CA, USA) in a pre-sintered state. The fabricated specimens were sintered according to manufacturer guidelines. The final dimensions were obtained with disk-shaped specimens having a 15-mm diameter and a 2-mm thickness after sintering. Then, the surface was uniformly polished using SiC abrasive paper up to 1200 grit. As outlined in Figure 1, the fabricated specimens were randomly divided into the following groups:

**As-sintered zirconia group**: The specimens were sintered and then surface-treated by acid etching. The etching solution comprised a mixture of 25% hydrofluoric acid, 16% sulfuric acid, hydrogen peroxide, methyl alcohol, and purified water. For the preparation of the etching solution, hydrofluoric acid: sulfuric acid: catalyst: methyl alcohol was mixed in a volume ratio of 7:5:1:2, and the preparation was completed by mixing the etchant composition and purified water at a ratio of 1:1. After heating the etching solution to 80 °C, surface treatment was performed on the zirconia specimen. This group was divided into 11 subgroups (*n* = 10 each) according to etching time (Control, 1, 2, 3, 5, 8, 10, 12, 15, 20, 30 min).

**Air-abraded zirconia group**: After sintering was completed, the specimens of this group were subjected to air abrasion at a distance of 10 mm and 0.3 MPa of pressure using aluminum oxide particles. The surface was treated by acid etching using the same etching solution. Air abrasion was applied to all specimens using the same technique. This group was likewise divided into 11 subgroups (*n* = 10 each) according to etching time (Control, 1, 2, 3, 5, 8, 10, 12, 15, 20, 30 min).

### 2.2. Surface Roughness Analysis

For each specimen group, data were measured (DektakXT, Bruker, Hamburg, Germany) five times to obtain the surface roughness data. The average of the data was obtained using calculation software based on the data peaks and valleys. The arithmetic mean (Ra) data were thereby obtained.

### 2.3. X-ray Diffraction (XRD) Analysis

The phase transformation by the surface treatment of each group was analyzed by measuring the peak intensity ratio of the zirconia specimens using a high-resolution X-ray diffractometer (HR-XRD, ATX-G, Rigaku Co., Kuraray, Japan) under Cu-Kα (1.54 Å) irradiation, 45 kV, 200 mA, 20–90°, and a 0.02° step size. The monoclinic peak intensity ratio (Xm) was calculated by the method described by Garvie and Nicholson [48], as follows:

$$\mathrm{Xm}=\frac{I_{m}\left(\overset{’}{1}11\right)+I_{m}\left(111\right)}{I_{m}\left(\overset{’}{1}11\right)+I_{m}\left(111\right)+I_{t}\left(111\right)}$$

where $I_{m}\left(\overset{’}{1}11\right)$ and $I_{m}\left(111\right)$ denote monoclinic peak intensities at 2θ values of 28.2° and 31.4°, respectively, and $I_{t}\left(111\right)$ represents the tetragonal peak intensity at 2θ = 31.1°. The volumetric content (Vm; %) of the monoclinic phase was calculated as follows:

$$\mathrm{Vm}=\frac{1.311Xm}{1+0.311Xm}$$

### 2.4. Scanning Electron Microscopy (SEM) Analysis

The surface morphology was observed and analyzed using field-emission scanning electron microscopy (FESEM, JEOL 7800F, Tokyo, Japan). The specimens were analyzed under high vacuum at 5 kV and observed at 20,000× magnification.

### 2.5. Biaxial Flexural Strength

In accordance with ISO 6872, biaxial flexural strength tests were performed on a universal testing machine (Instron 3366, Instron Corp., Norwood, MA, USA) using the piston-on-three-ball technique. Three 5-mm-diameter stainless steel balls were arranged to form a support circle with a diameter of 12 mm and an angle of 120° to each other. The specimen was placed exactly in the center. The load piston was applied (1.4-mm diameter) at a crosshead speed of 1.0 mm/min. The fracture load of each specimen was measured, and the biaxial flexural strength was calculated according to the following formula:

$$\mathrm{FS}=\frac{-0.2387\;P\left(X-Y\right)}{h^{2}}$$

Here, FS denotes biaxial flexural strength (MPa), *P* is the fracture load (N), and *h* represents the thickness of the disk at the fracture origin (mm). *X* and *Y* are determined by the following formula:

$$X=\left(1+u\right)\ln{\left(\frac{r2}{r3}\right)}^{2}+\left[\frac{1-u}{2}\right]{\left(\frac{r2}{r3}\right)}^{2}\;Y=\left(1+u\right)\left[1+\ln{\left(\frac{r1}{r3}\right)}^{2}\right]+\left(1-u\right){\left(\frac{r1}{r3}\right)}^{2}$$

where *u* is Poisson’s ratio (0.25), *r*1 is the radius of the support circle, *r*2 is the radius of the load piston, and *r*3 is the specimen radius.

The Weibull characteristic strength (*σ*_0_) and Weibull modulus (*m*) were calculated according to Equation:

$$P_{f}=1-exp\left[-{\left(\frac{\sigma }{\sigma_{0}}\right)}^{m}\right]$$

where *P_f_* is the probability of failure (between zero and one), *σ* is the flexural strength in MPa, and *σ*_0_ is the Weibull characteristic strength in MPa (63.2% of specimen failure).

### 2.6. Evaluation of Weight Change of Zirconia Specimens

The weight change before and after the air-abrasion and etching process was measured and evaluated. The fabricated specimens were sintered according to manufacturer guidelines. The final dimensions were obtained with disk-shaped specimens having a 10-mm diameter and a 2-mm thickness after sintering. Then, the surface was uniformly polished using SiC abrasive paper up to 1200 grit. In the as-sintered zirconia group, the initial weight was measured after preparation of the specimen, and in the air-abraded zirconia group, the weight was measured after applying aluminum oxide particles at a distance of 10 mm at a pressure of 0.3 MPa. All specimens were etched for 30 min using an etching solution, dried, and then weighed again. The rate of change in weight for each specimen was calculated as follows:

$$\frac{\left[\left(\mathrm{Initial}\;\mathrm{weight}\right)-\left(\mathrm{Weight}\;\mathrm{after}\;\mathrm{acid}\;\mathrm{treatment}\right)\right]}{\left(\mathrm{Initial}\;\mathrm{weight}\right)}\times 100\;\left(\%\right)$$

### 2.7. Statistical Analysis

Shapiro–Wilk’s test and Levene’s test were performed to confirm data normality and variance homogeneity. Surface roughness and biaxial flexural strength values were analyzed using one-way ANOVA and the post-hoc Tukey–Kramer test (α = 0.05). A paired *t*-test was used to evaluate the weight change before and after the air-abrasion and etching process (α = 0.05). All statistics were analyzed using statistical analysis software (SPSS v25.0, IBM, Armonk, NY, USA).

## 3. Results

### 3.1. SEM Analysis and Surface Roughness Analysis

The zirconia surface roughness data following the pretreatment method were obtained using surface profiler equipment, as shown in Table 1. For the as-sintered zirconia specimens, the roughness increase was not clearly apparent when the etching time was less than 3 min. However, the roughness increased significantly in the as-sintered zirconia samples subjected to etching for 5 min compared to the base measuring time point (control; *p* < 0.001). After 15 min, the roughness was approximately 0.2 µm, which is similar to that of the surface after air abrasion. In the air-abraded zirconia specimens, there was no significant change in roughness at the beginning of the application of the etching solution. However, the roughness increased after 10 min (*p* = 0.016) and showed a roughness average (Ra) value of more than 0.3 µm after 20 min (*p* = 0.016). The roughness of the air-abraded zirconia samples was high at all times. It was confirmed that the Rz value also showed a tendency to increase as the etching time increased, similar to Ra.

**Figure 2:**
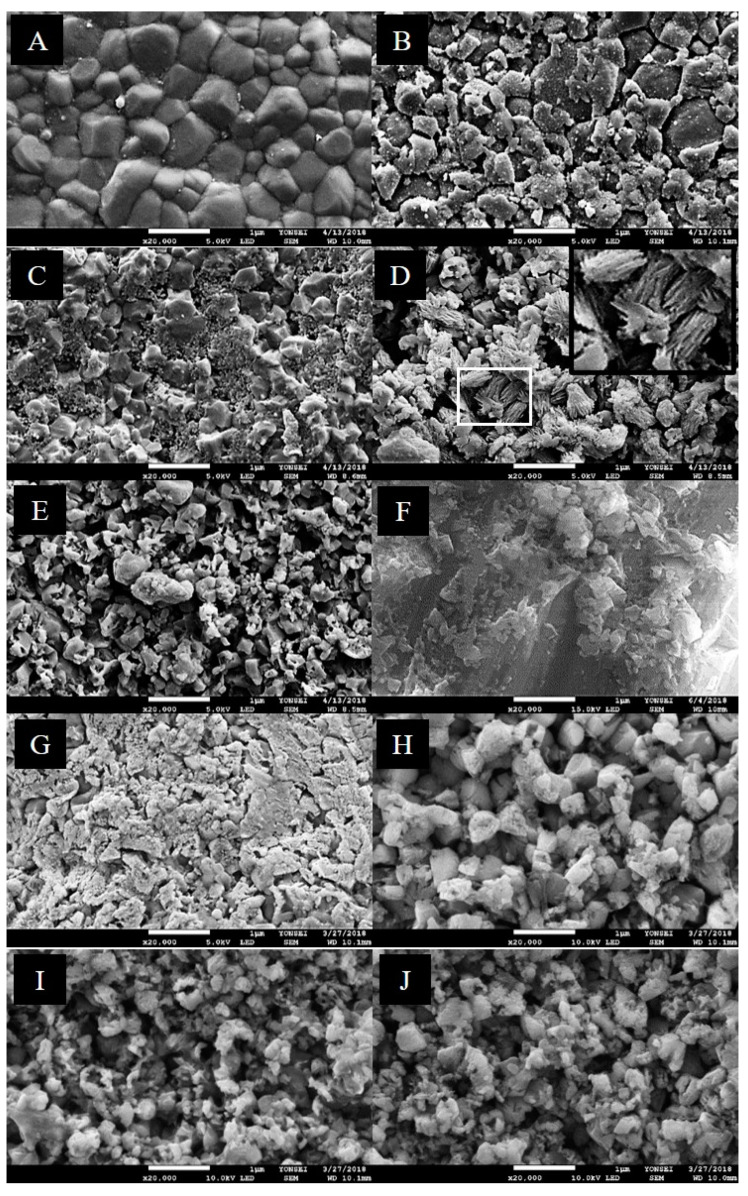
SEM micrographs (magnification 20,000×) of as-sintered and air-abraded zirconia specimens following etching for different time periods. As the etching time increased, the grain size decreased and became irregular overall. (**A**) As-sintered zirconia surface. (**B**–**E**) As-sintered zirconia surface subjected to etching for 1, 5, 15, and 30 min. (**F**) Air-abraded zirconia surface. (**G**–**J**) Air-abraded zirconia surface subjected to etching for 1, 5, 15, and 30 min. The surface of air-abraded zirconia was rougher and more irregular. (**D**) The domain structure due to the transformation intothe monoclinic zirconium dioxide (black square).

Various findings were observed in the SEM images. In the control as-sintered and air-abraded zirconia samples (Figure 2A,F), the surface difference with and without air abrasion can be observed. Nevertheless, the roughness significantly increased as the etching application time increased. Following the etching of the as-sintered zirconia specimen for 1 min (Figure 2B), the existing zirconia crystal structures were observed. However, dissolution of the crystal structure connection site owing to the etching process was confirmed. At 5 min after the etching application (Figure 2C), some inherent zirconia crystal structures were observed. It was evident that the surface roughness due to etching on the crystal structure increased greatly. In the specimens subjected to etching for a longer time, the surface change was so significant that the shape of the existing crystal structure was scarcely recognized, and the roughness increased. Domain structures of transformed monoclinic ZrO_2_ were observed on the as-sintered zirconia surface (Figure 2D) after etching for 15 min. For the air-abraded zirconia specimen subjected to etching for 5 min (Figure 2H), the cracking and loss of a superficial-grain crystal structure were much more pronounced than those in the 5-min-etched as-sintered zirconia specimen (Figure 2C). As a result, it was confirmed that the overall surface of the air-abraded specimens is more irregular.

### 3.2. Phase Transformation

**Figure 3:**
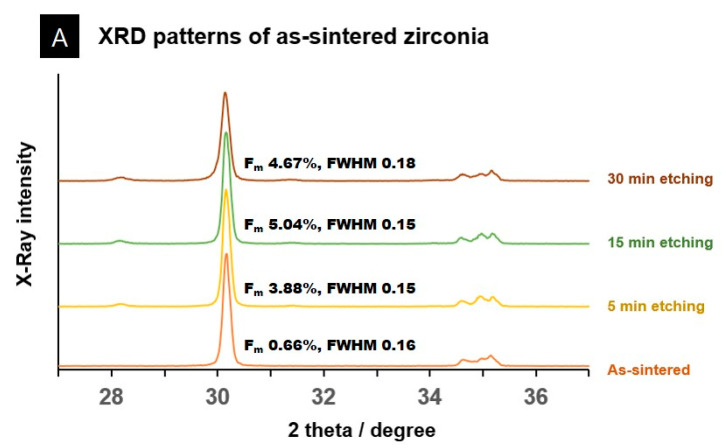

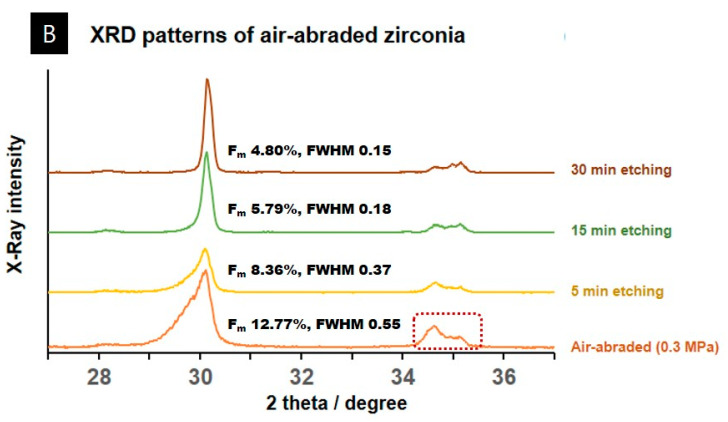
XRD patterns of as-sintered and air-abraded zirconia specimens. (**A**) As-sintered zirconia group. A slight increase in the monoclinic phase was visible; FWHM did not show a significant change. (**B**) Air-abraded zirconia group. Immediately after air-abrasion treatment, an increase of monoclinic phase was seen (orange colored line); an increase in the FWHM and reversed intensity of peaks (002)_t_ and (110)_t_ in the region of 2θ = 30° were observed (red dotted box). However, as the etching time increased, the monoclinic phase and FWHM decreased, and the reversed intensity of peaks also disappeared.

Figure 3 depicts XRD patterns of the as-sintered and air-abraded zirconia. In the as-sintered zirconia specimens, the t-m phase transformation occurred at 2θ = 28.2° as the etching time is increased (Figure 3A). In the air-abraded zirconia control specimens, the characteristic feature of the tetragonal peak (111)_t_ full width at half maximum (FWHM) in the region of 2θ = 30° significantly increased to 0.55. The FWHM shows a unique finding, i.e., the width gradually decreased with an increase in the etching time.

The air-abraded zirconia control specimens showed a reversed intensity of peaks (002)_t_ and (110)_t_. However, as the etching time increased, they recovered to the same shape as those without air abrasion (Figure 3B). With respect to the monoclinic-phase content ratio of the as-sintered zirconia group, a ratio increase of 1.97–5.47% occurred as the etching time increased. In the case of air-abraded zirconia, the ratio increased rapidly to 12.77% immediately after air abrasion, whereas the monoclinic-phase content gradually decreased as the etching time increased (Table 2). After 12 min of etching in the air-abraded zirconia group, the monoclinic phase reduced by 4.09–5.86%, which is similar to that of the as-sintered specimens.

The XRD pattern with diffraction degree ranging from 20 to 90 degrees and the XRD pattern with the y-axis scale changed to logarithmic are presented in the supplement information (Supplementary Material Figures S1–S3).

### 3.3. Biaxial Flexural Strength

**Figure 4:**
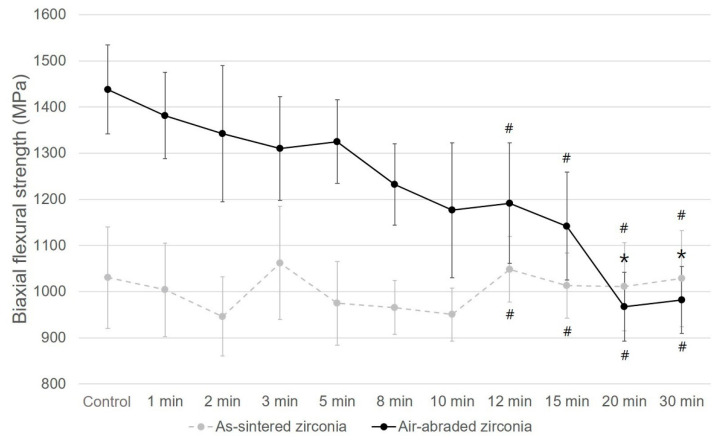
Biaxial flexural strength (mean ± standard deviation) of as-sintered and air-abraded zirconia in accordance with etching time (*n* = 10 per timepoint, MPa). The asterisk indicates significance compared to the control specimen of air-abraded zirconia (*p* < 0.05). The as-sintered zirconia group showed no significant difference in biaxial flexural strength with etching time (*p* > 0.05). The pound symbol indicates no significant difference between the as-sintered and air-abraded zirconia samples at the same etching time (*p* > 0.05).

The biaxial flexural strengths of the as-sintered and air-abraded zirconia groups in accordance with etching times are shown in Figure 4. In the as-sintered zirconia case, a flexural strength of 1030.4 MPa was seen in the group of samples that were not subjected to etching. A slight change was evident in the flexural strength in accordance with the etching time; however, this was not statistically significant at any time point (*p* = 0.086). A flexural strength in the range of 946.4–1062.0 MPa was evident.

The air-abraded zirconia samples that were not subjected to etching showed a high flexural strength of 1438.1 MPa. However, as the etching time increased, the flexural strength gradually decreased. The flexural strength of the air-abraded zirconia samples subjected to etching for more than 12 min showed no significant difference compared to that of the as-sintered zirconia samples subjected to etching for 12 min.

In the as-sintered zirconia group, there was no significant correlation between the Weibull modulus (m) and Weibull characteristic strength (σ0) according to the etching time. However, in the air-abraded zirconia group, the Weibull modulus increased in the 20 and 30 min etching groups, and the Weibull characteristic strength (σ0) value continuously decreased with the etching time (Table 3).

### 3.4. Weight Change of Zirconia Specimens

**Figure 5:**
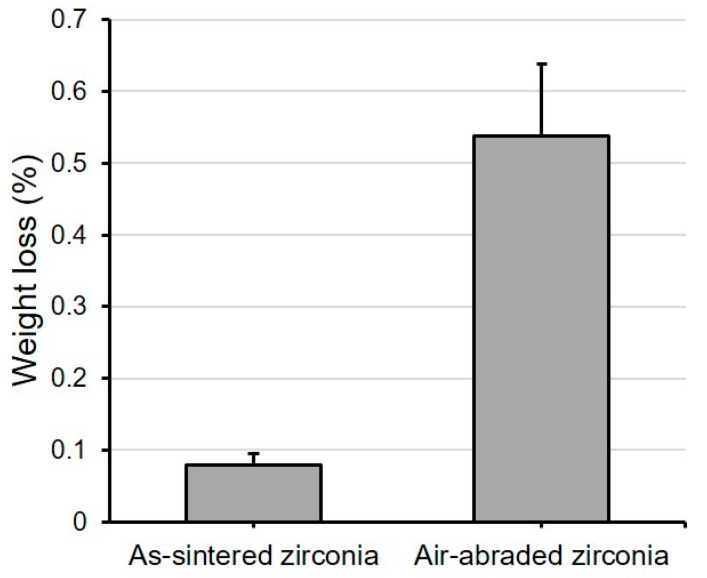
The weights of as-sintered zirconia and air-abraded zirconia were evaluated before and after the etching process. In the air-abraded zirconia group, the weight after etching decreased significantly about 0.54% compared to the 0.08% loss in the as-sintered group (*p* < 0.001).

The weights of as-sintered zirconia and air-abraded zirconia were evaluated before and after the etching process (Figure 5). Before etching the zirconia specimens, both the as-sintered zirconia and air-abraded zirconia groups showed an average weight of about 934.4 mg. However, after acid treatment, only about 0.08% weight loss occurred in the as-sintered zirconia group, while a large change of 0.54% occurred in the air-abraded zirconia group (*p* < 0.001).

## 4. Discussion

In this study, the effects of acid mixtures containing hydrofluoric acid, sulfuric acid, hydrogen peroxide, and other compounds on the surface roughness, phase transformation, and biaxial flexural strength of as-sintered and air-abraded 3Y-TZP were evaluated. The etching process using acid mixtures increased the surface roughness of both as-sintered and air-abraded zirconia groups. However, the as-sintered zirconia samples showed a slight increase of monoclinic-phase contents, even when the etching time was increased. On the other hand, in the air-abraded zirconia group, the monoclinic phase was greatly increased owing to the t-m phase transformation after air abrasion, and it decreased during the etching process. Similarly, in the case of the as-sintered zirconia samples, the biaxial flexural strength did not change significantly as the etching time increased. On the other hand, in the air-abraded zirconia group, the biaxial flexural strength markedly increased immediately after air abrasion. After 20 min of etching, the flexural strength decreased significantly compared to the samples subjected to air-abrasion only. These results show that the hypothesis of this study was rejected.

### 4.1. SEM Analysis and Surface Roughness Analysis

The SEM results of the surface treatment method and etching time showed that after acid treatment, the grains of the zirconia samples became highly irregular and the grain size decreased for both the as-sintered and air-abraded zirconia specimens. Since the crystal boundary was more chemically reactive and dissolved faster, it is believed that irregular grooves formed around the crystal and the grain size was reduced [37]. Moreover, as suggested in the study of Alhassan et al. [47], the addition of sulfuric acid may also contribute to crystal size reduction. In that study, the crystal size decreased as the weight ratio of sulfate increased [47]. Needle-like structures were observed in the as-sintered zirconia after etching for 15 min, which has been reported as a combination of zirconium, yttrium, and fluorine [24]. In the case of air-abraded zirconia, the surface irregularity was slightly higher because the air-abrasion process was performed first, causing surface roughness and creating more space for the penetration of the etching solution.

The roughness of the zirconia specimens continued to increase in both as-sintered and air-abraded zirconia. However, it is known that sulfuric acid or hydrogen peroxide among the components of the acid mixture does not significantly increase the roughness [49,50]. Previous studies also analyzed that the mixture of sulfuric acid and hydrogen peroxide, also known as the Piranha solution, did not contribute to the increase in roughness and increase in bond strength [49,50]. It is thought that hydrofluoric acid among the ingredients used in this study contributed to the increase in roughness.

### 4.2. XRD

The XRD findings of the air-abraded zirconia control group in this study corroborated the findings of previous studies that reported the three characteristics that appear after the air-abrasion surface treatment of zirconia samples [51]: first, an increase in the monoclinic phase occurred in the 2θ = 28.2° region; second, the tetragonal peak (111)_t_ broadened in the 2θ = 30° region; finally, a reversal in the intensities of peaks (002)_t_ and (110)_t_ was observed. In addition, the monoclinic phase volume increased by 12.09%.

However, as the etching time increased in the air-abraded zirconia specimen, the monoclinic phase contents decreased, the FWHM decreased in tetragonal peak (111)_t_, and the peak reversal disappeared in (002)_t_ and (110)_t_ regions, all of which were unique features not previously observed in other studies. Similar findings of regeneration firing, i.e., reverse transformation, were reported in previous studies. Regeneration firing was primarily observed when thermal annealing was performed in furnaces at temperatures above 900 °C for manufacturing zirconia [23,52]. The regeneration firing process reduced the compressive stress and increased the tetragonal phase content [23,52]. Similar phenomena have been reported in studies of grinding zirconia surfaces. Moreover, the very high heat generated during the grinding process caused reverse transformation [53]. In the present study, no high-temperature annealing process was used as the regeneration firing process. In this study, the decrease in the monoclinic phase when the etching process was performed after air-abrasion appears to be due to the result that loose surface particles in the monoclinic phase generated through air-abrasion were removed as the etching process proceeded for 30 min. This can be confirmed by a significant decrease in the weight after etching in the air-abraded zirconia group. This may also have been attributed to the loss of monoclinic zirconia grains on account of the longer acid treatment time and the remaining of yttrium grains, which are relatively resistant to loss. It is surmised that a monoclinic phase decrease and a tetragonal phase increase occurred for this reason [23].

On the other hand, Ding et al. [39] showed that the monoclinic volume of as-sintered zirconia samples was 15.68% and reported a slight increase in the monoclinic volume (16.89%) in zirconia samples subjected only to air-abrasion. In the zirconia samples treated with 40% hydrofluoric acid for 60 min after air abrasion, the monoclinic volume increased significantly to 39.14%. Their findings are in contrast with the results of the present study, in which the monoclinic volume greatly increased during air abrasion and gradually decreased during the etching process. In general, a monoclinic phase increase is associated with increased susceptibility to surface cracks, low-temperature degradation, and lower reliability [52,54,55].

In the as-sintered zirconia group, a monoclinic volume of 3.08–6.02% was observed depending on the etching time. Nonetheless, it was not statistically significant and was very small compared to the values reported in other studies. This result is very similar to that of Xie et al. [45], who reported that no monoclinic phase volume was observed in any group treated with hydrofluoric acid or acetic acid, except for the group subjected to hydrothermal aging under pressure at 134 °C and 0.2 MPa. On the other hand, Flamant et al. [24] reported that hydrofluoric acid etching on zirconia specimens resulted in the loss of yttrium oxide and zirconia oxide, which increased the monoclinic phase after acid treatment because it made the tetragonal phase unstable. This finding is contrary to the results of the present study.

### 4.3. Biaxial Flexural Strength

Various studies were conducted on the effect of air abrasion on the zirconia flexural strength with alumina oxide particles. Chintapalli et al. [41] reported that 110–120-µm Al_2_O_3_ particles significantly increased the Y-TZP flexural strength through transformation toughening. In addition, many researchers have published studies supporting the increase in flexural strength when air abrasion is performed on zirconia surfaces [4,40,42]. In the present study, the control group without etching in air-abraded zirconia specimens showed the highest flexural strength, demonstrating the effect of t-m phase transformation, which was the same conclusion drawn in many previous studies.

On the other hand, the control group of air-abraded zirconia showed the highest flexural strength, which then gradually decreased as the etching progressed. Iijima et al. [43] similarly reported that the roughness increased on account of acid treatment after air abrasion; however, the flexural strength decreased. In general, it is known that the final flexural strength is determined by the balance point between the flexural strength increase factor through the surface treatment toughening mechanism and the decrease factor due to critical defects [52]. This study also showed the highest flexural strength of the control group of air-abraded zirconia; then, the flexural strength decreased in accordance with the increased etching time. This may have been due to the formation of critical defects from advanced cracks [23,43,44,52].

In addition, the hydroxyl (OH) group generated by treating hydrofluoric acid with zirconia could penetrate the Y-TZP surface stress zone to increase lattice spacing and accelerate low-temperature degradation (LTD) [43]. In this study, the hydrogen peroxide contained in the acid mixture may have had a greater influence on the formation of OH groups in addition to the acid, such as hydrofluoric acid, which may have contributed to the decreased strength. In the case of as-sintered zirconia, although the surface roughness increased as the etching time increased, the flexural strength was not significantly affected.

In this study, the effects of acid mixture on the as-sintered and air-abraded 3Y-TZP surfaces were investigated in terms of respective topography, XRD, and biaxial flexural strength analyses of the surfaces. Surface roughness increased in accordance with etching time in all groups. In the XRD and biaxial flexural strength analyses, the etching effect on the as-sintered zirconia was weak, while the monoclinic phase contents and flexural strength decreased for the air-abraded zirconia. Nevertheless, this study has some limitations. Because only one acid-mixture combination was used in this study, and the zirconia surface was not analyzed based on the acid concentration difference or acid-mixing ratio. In addition, the effects of LTD through the aging process and acid etching for longer than 30 min were not confirmed. To confirm the long-term clinical performance of the zirconia prosthesis and to reach a more definitive conclusion, it is necessary to study the effects of LTD and to analyze the effects of various parameters of the acid mixture and longer acid treatment.

## 5. Conclusions

Considering the study limitations described above, the following conclusions were drawn:

1. Surface treatment using acid mixture on both as-sintered and air-abraded 3Y-TZP surfaces further increased the surface roughness.
2. Surface treatment using acid mixture of the as-sintered zirconia surface produced a slight increase of the monoclinic-phase contents. On the surface of air-abraded zirconia, the monoclinic phase content increased considerably after air abrasion; however, the removal of loose monoclinic particles was occurred with the increase in acid treatment time.
3. Surface treatment using acid mixture of the as-sintered zirconia surface did not significantly affect the biaxial flexural strength. The flexural strength of the air-abraded zirconia surface increased significantly; however, as the acid treatment time increased, the biaxial flexural strength gradually decreased to almost the same strength as that of the untreated surface.

Therefore, it was confirmed that surface treatment of as-sintered zirconia and air-abraded zirconia samples with acid mixtures increased the surface roughness of the zirconia samples without adversely affecting their flexural strength. Surface treatment with acid mixtures may thus be helpful in clinical applications involving the use of zirconia surfaces with luting agents or veneered ceramics.

## Figures and Tables

**Table 1:** Surface roughness (Ra, Rz) value (mean ± standard deviation) for as-sintered and air-abraded zirconia surfaces (*n* = 5 per timepoint, µm). The change in roughness with etching time is confirmed, and the roughness of both samples gradually increased with time. The asterisk indicates significance compared to the corresponding control specimen of the as-sintered and air-abraded zirconia (*p* < 0.05).

Etching Time	Ra (μm)		Rz (μm)	
	As-Sintered Zirconia	Air-Abraded Zirconia	As-Sintered Zirconia	Air-Abraded Zirconia
Control	0.048 (0.009)	0.202 (0.026)	0.392 (0.029)	0.773 (0.141)
1 min	0.077 (0.015)	0.218 (0.049)	0.502 (0.083)	1.054 (0.223)
2 min	0.071 (0.009)	0.23 (0.034)	0.476 (0.053)	1.271 (0.159)
3 min	0.073 (0.01)	0.2 (0.023)	0.577 (0.065)	0.954 (0.157)
5 min	0.151 (0.026) *	0.211 (0.019)	0.644 (0.083)	1.164 (0.237)
8 min	0.19 (0.022) *	0.264 (0.031)	1.159 (0.151) *	1.108 (0.273)
10 min	0.174 (0.022) *	0.281 (0.034) *	1.122 (0.285) *	1.056 (0.224)
12 min	0.185 (0.039) *	0.278 (0.032) *	1.122 (0.285) *	1.237 (0.15) *
15 min	0.203 (0.025) *	0.286 (0.033) *	1.189 (0.274) *	1.221 (0.12) *
20 min	0.198 (0.026) *	0.31 (0.036) *	1.091 (0.29) *	1.389 (0.113) *
30 min	0.211 (0.018) *	0.3 (0.034) *	1.332 (0.218) *	1.364 (0.167) *

*R_a_, Roughness average. *R*_z_, Average maximum height of the profile.

**Table 2:** Monoclinic phase contents of the as-sintered and air-abraded zirconia samples in accordance with etching time. In as-sinter zirconia, the value slightly increased as the etching time increased, and in air-abraded zirconia, the value increased rapidly after air-abrasion, and then gradually decreased as the etching time increased.

Etching Time	As-Sintered Zirconia	Air-Abraded Zirconia
Control	0.58 (0.16)	13.16 (2.05)
1 min	2.05 (0.48)	12.5 (2.35)
2 min	3.87 (0.41)	10.59 (1.73)
3 min	3.65 (0.40)	8.35 (1.84)
5 min	3.93 (0.39)	8.51 (1.56)
8 min	4.01 (0.63)	8.05 (1.81)
10 min	4.71 (0.55)	7.57 (1.36)
12 min	4.33 (0.74)	5.68 (1.25)
15 min	4.89 (1.18)	5.76 (1.44)
20 min	5.11 (0.78)	4.12 (0.98)
30 min	4.92 (0.85)	3.73 (0.49)

**Table 3:** Mean and standard deviation of flexural strength and Weibull parameters of as-sintered and air-abraded zirconia specimens according to etching treatment time.

Etching Time		Flexural Strength (MPa)	*m* (95% CI)	σ_0_ (MPa)
As-sintered zirconia	Control	1030.4 (110.2)	10.3 (8.4–12.3)	1080.8
	1 min	1004.3 (101.3)	11.8 (9–14.5)	1047.6
	2 min	946.4 (85.6)	13.4 (8.9–17.9)	982.8
	3 min	1062 (122.5)	10.2 (7.5–12.9)	1114.3
	5 min	974.8 (90.5)	12.9 (7.7–18)	1014.0
	8 min	965.8 (58.7)	19.4 (16.2–22.6)	991.8
	10 min	950.5 (57.2)	21.6 (14.7–28.4)	973.8
	12 min	1048.4 (70.9)	17.6 (15–20.3)	1079.4
	15 min	1013.3 (70.7)	16.6 (11.8–21.4)	1045.1
	20 min	1010.8 (95.2)	12.5 (9.5–15.4)	1052.2
	30 min	1028.2 (104.5)	10.3 (7.3–13.4)	1078.8
Air-abraded zirconia	Control	1438.1 (96.2)	8.9 (6.9–10.9)	1517.7
	1 min	1381.6 (93.2)	8.3 (7.1–9.5)	1463.0
	2 min	1342.1 (147.7)	5.4 (4.5–6.2)	1454.4
	3 min	1309.7 (112.5)	7.4 (4.1–10.8)	1397.0
	5 min	1325.2 (90.4)	9.9 (4.9–14.9)	1393.6
	8 min	1232.4 (87.8)	9.4 (4.5–14.4)	1299.2
	10 min	1176.4 (146.2)	5.1 (1.8–8.5)	1286.7
	12 min	1191.7 (130.4)	4.9 (3.4–6.4)	1304.8
	15 min	1141.7 (116.8)	5.8 (4.7–6.9)	1231.6
	20 min	967.3 (74.2)	16.7 (10.4–23)	997.8
	30 min	981.9 (72.9)	17.1 (10.1–24.1)	1012.2

## Data Availability

The data presented in this study are available on request from the corresponding author.

## Supplementary Materials

The following are available online at https://www.mdpi.com/article/10.3390/ma14092359/s1, Figure S1. XRD patterns of as-sintered and air-abraded zirconia specimens with diffraction degree from 20 to 90 degrees. Figure S2. The XRD pattern of as-sintered zirconia group in which the diffraction degree is from 20 to 90 degrees and the y-axis scale is changed to logarithmic. Figure S3. The XRD pattern of air-abraded zirconia group in which the diffraction degree is from 20 to 90 degrees and the y-axis scale is changed to logarithmic.
