# Supplementary material for: Effect of Acid Mixtures on Surface Properties and Biaxial Flexural Strength of As-Sintered and Air-Abraded Zirconia
Source: Materials (Basel). 2021 May 1;14(9):2359. doi: 10.3390/ma14092359 (PMC8125152; doi:10.3390/ma14092359)
Supplement: Supplementary file 1 [file materials-14-02359-s001.zip › materials-1122648-supplementary.pdf]

materials

MDPI

Supplementary Materials: Effect of Acid Mixtures on Surface Properties and Biaxial Flexural Strength of As-Sintered and Air-Abraded Zirconia

Jong-Eun Kim, Yong-Chan Kwon, Sunjai Kim, Young-Bum Park, June-Sung Shim and Hong-Seok Moon*

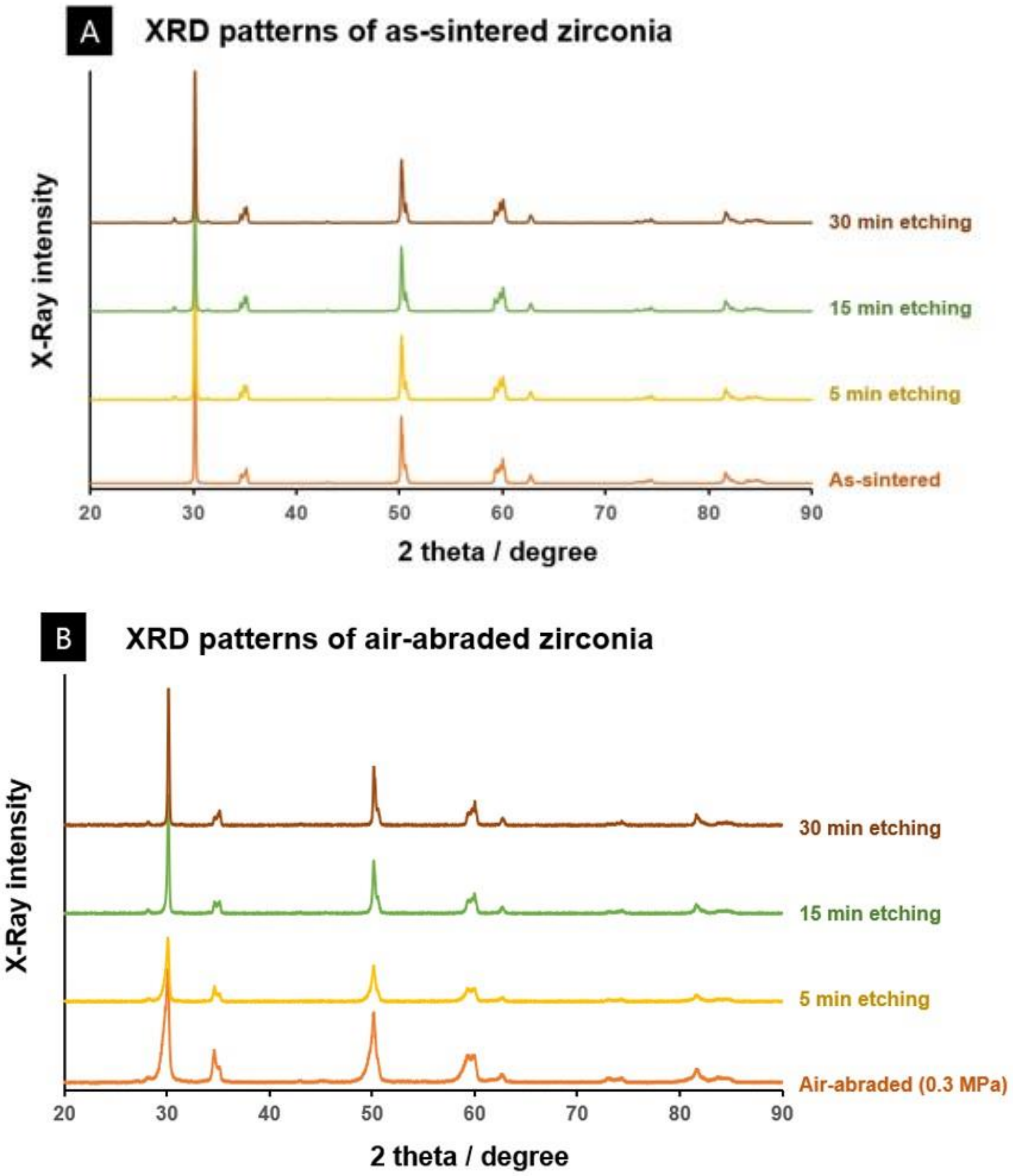

Figure S1. XRD patterns of as-sintered and air-abraded zirconia specimens with diffraction degree from 20 to 90 degrees. (**A**) As-sintered zirconia group. (**B**) Air-abraded zirconia group.

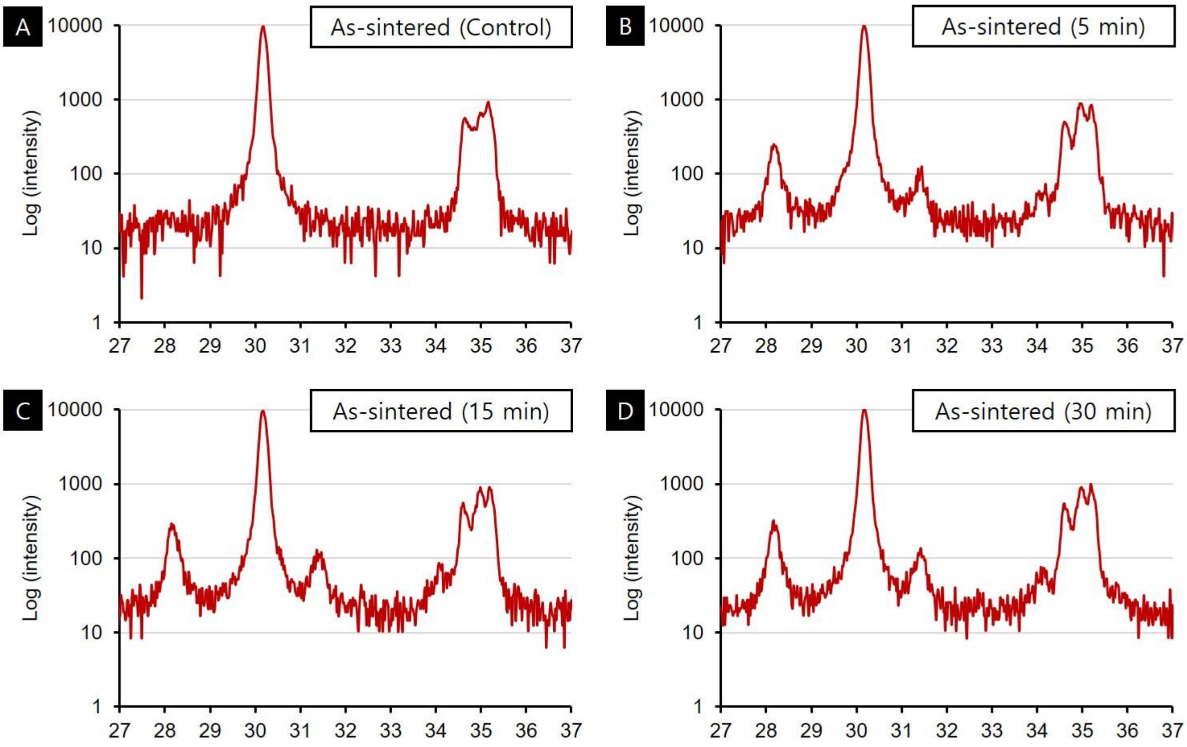

Figure S2. The XRD pattern of as-sintered zirconia group in which the diffraction degree is from 20 to 90 degrees and the y-axis scale is changed to logarithmic. (A) control group. (B) 5 min etching group. (C) 15 min etching group. (D) 30 min etching group.

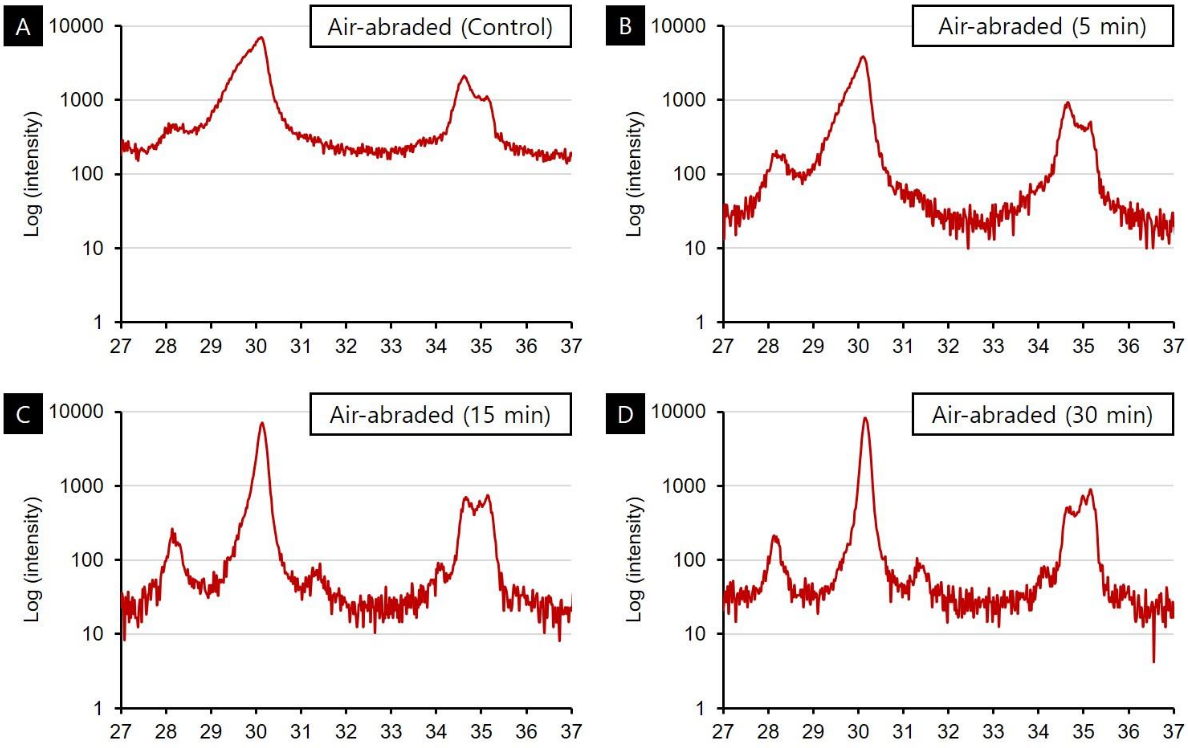

Figure S3. The XRD pattern of air-abraded zirconia group in which the diffraction degree is from 20 to 90 degrees and the y-axis scale is changed to logarithmic. (**A**) control group. (**B**) 5 min etching group. (**C**) 15 min etching group. (**D**) 30 min etching group.
